# Supplementary material for: Genome-Wide Profile of Mutations Induced by Carbon Ion Beam Irradiation of Dehulled Rice Seeds
Source: Int J Mol Sci. 2024 May 10;25(10):5195. doi: 10.3390/ijms25105195 (PMC11121050; doi:10.3390/ijms25105195)
Supplement: Supplementary file 1 [file ijms-25-05195-s001.zip › ijms-2961226-supplementary.pdf]

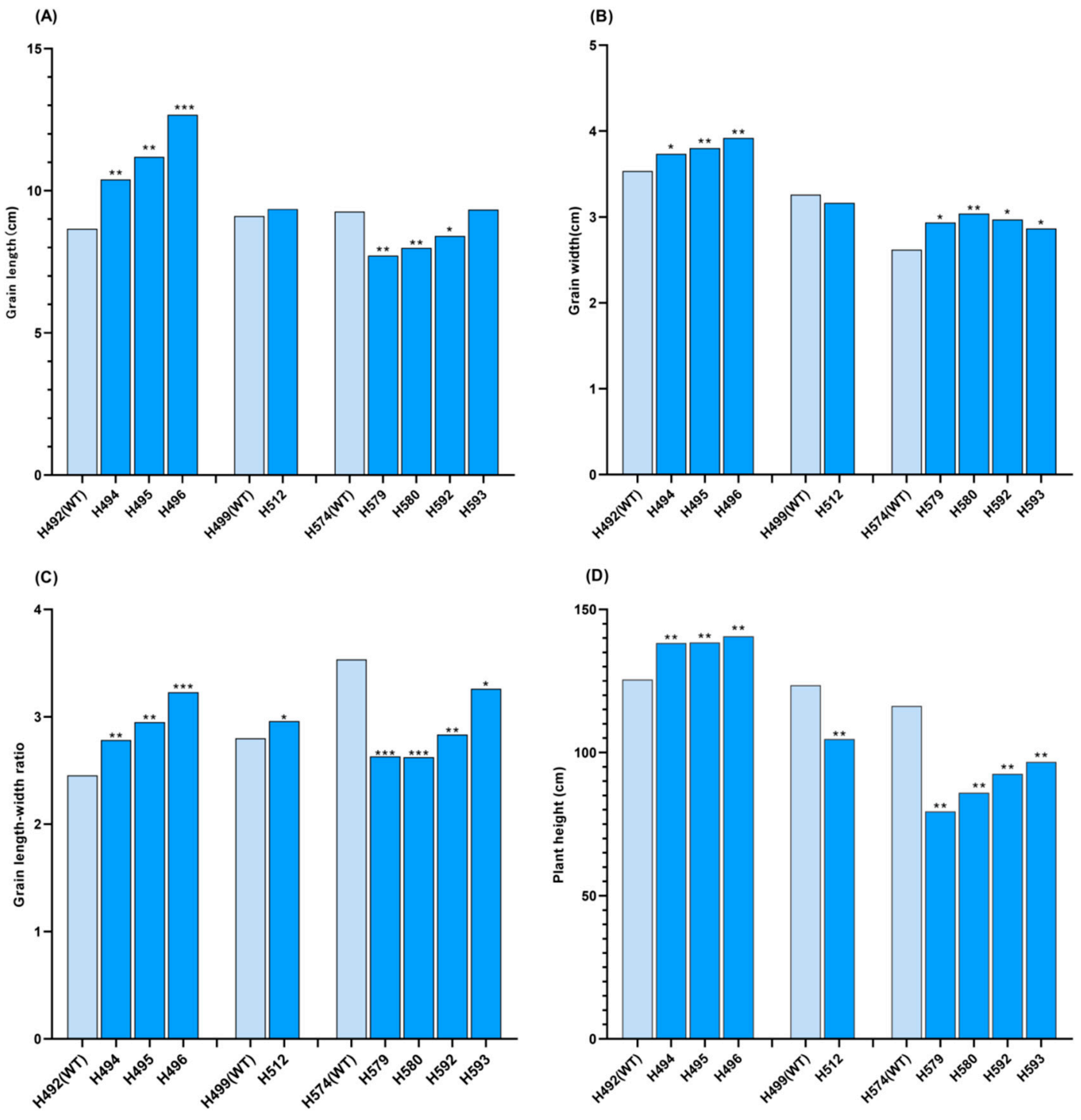

Figure S1. Comparison of agronomic traits between wild types and mutants. (A) Variations in grain length; (B) Variations in grain width; (C) Variations in grain length-width ratio; (D) Variations in plant height.

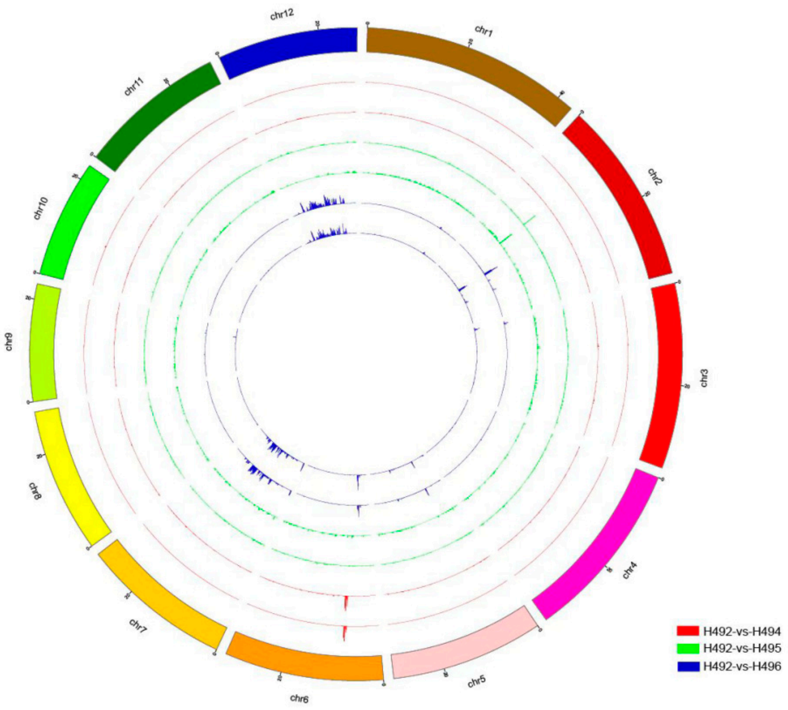

Figure S2. Distribution of mutation sites of H492 mutant on chromosome.Inner ring: InDel; Outer ring: SBS.

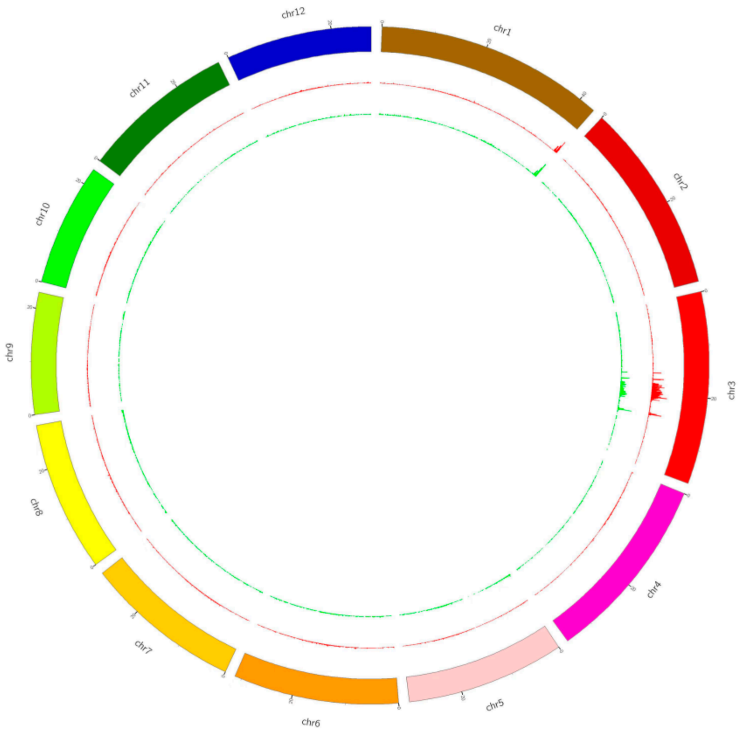

Figure S3. Distribution of mutation sites of H499 mutant on chromosome, inner ring: InDel; Outer ring: SBS.

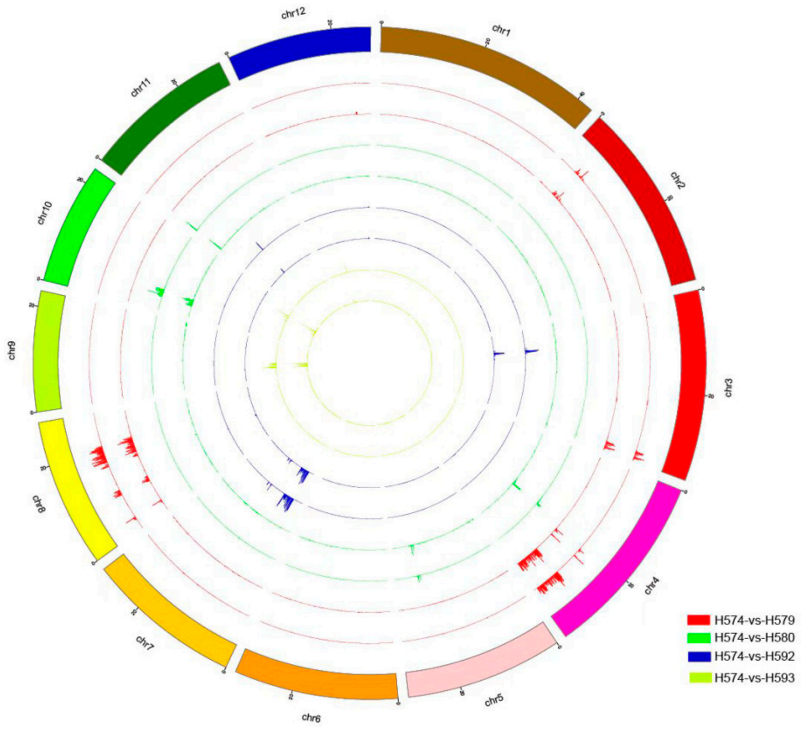

Figure S4. Distribution of mutation sites of H574 mutant on chromosome. Inner ring: InDel；Outer ring: SBS.

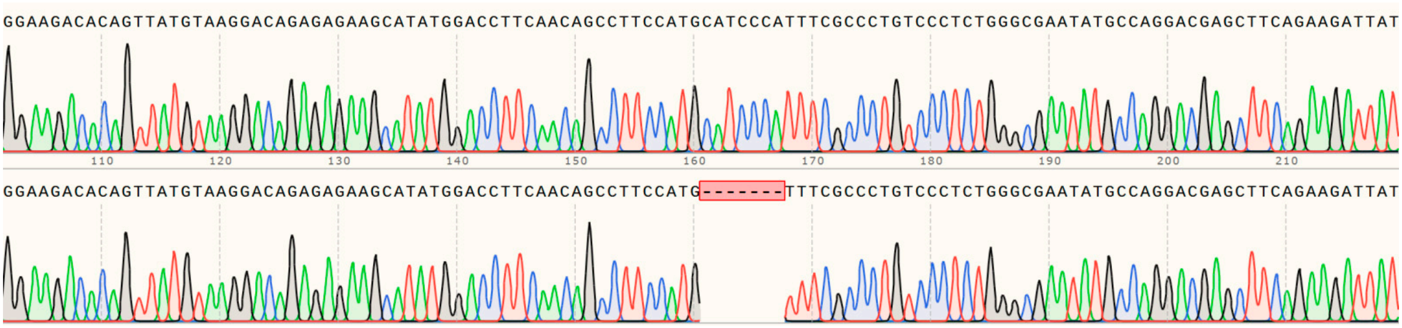

Figure S5. Comparing the sequences of *Os03g0576600* of H499 (WT) and H512. The deletion occurred at the 21,083,265 bp on chromosome 3.

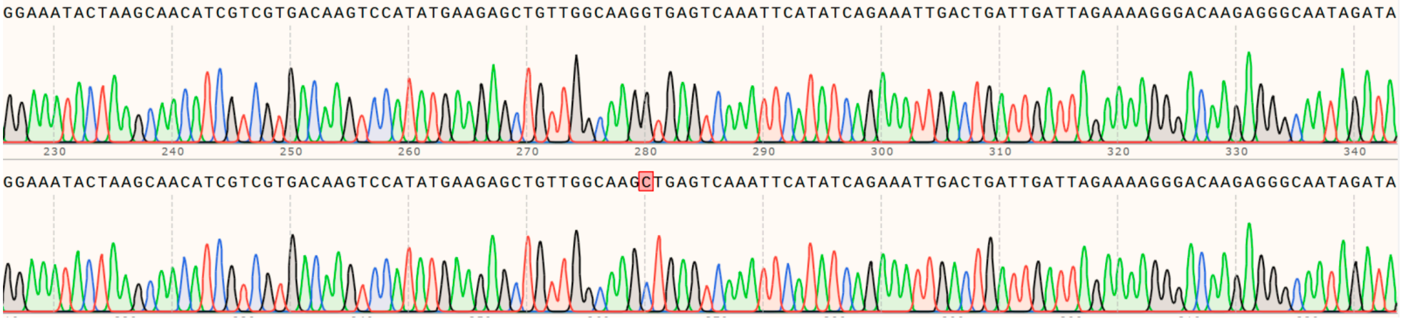

Figure S6. Comparing the sequences of *Os03g0576600* of H499 (WT) and H512. The SBS occurred at the 21,083,384 bp on chromosome 3.

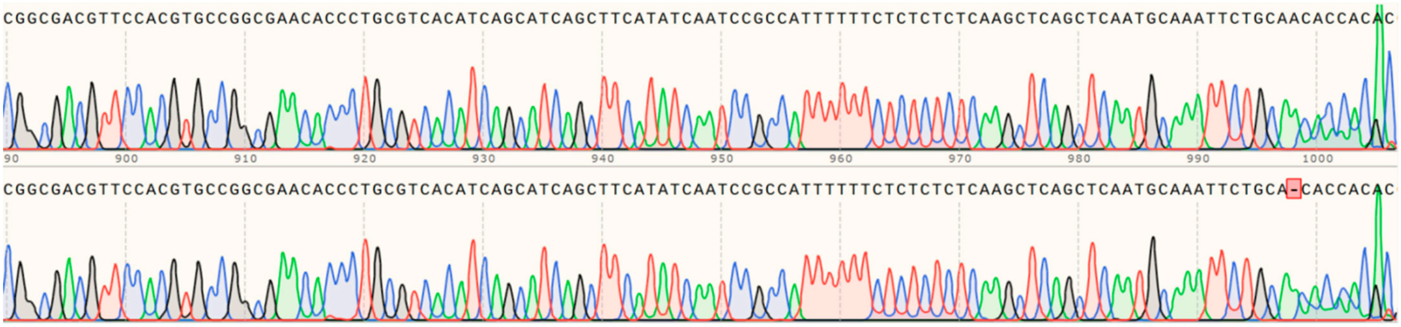

Figure S7. Comparing the sequences of *Os08g0425500* of H574 (WT) and H579. The deletion occurred at the 20,451,690 bp on chromosome 8.

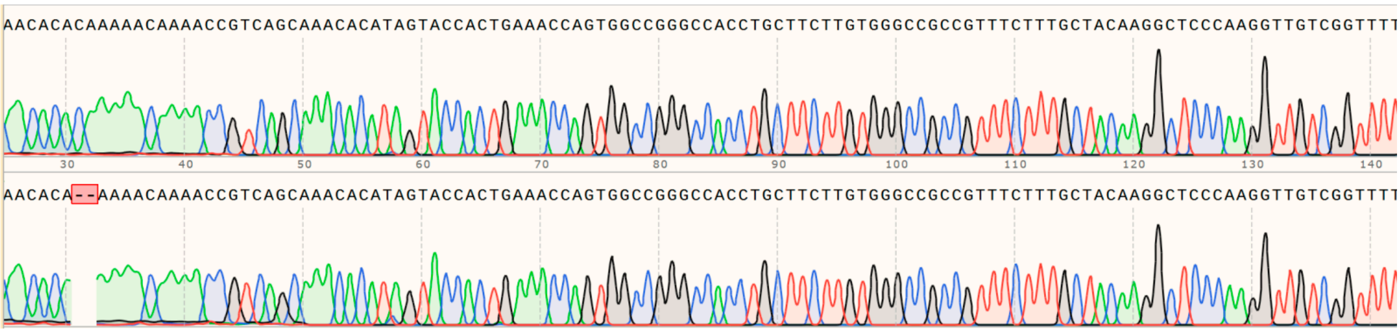

Figure S8. Comparing the sequences of *Os11g0112050* of H574 (WT) and H580. The deletion occurred at the 564,027 bp on chromosome 11.

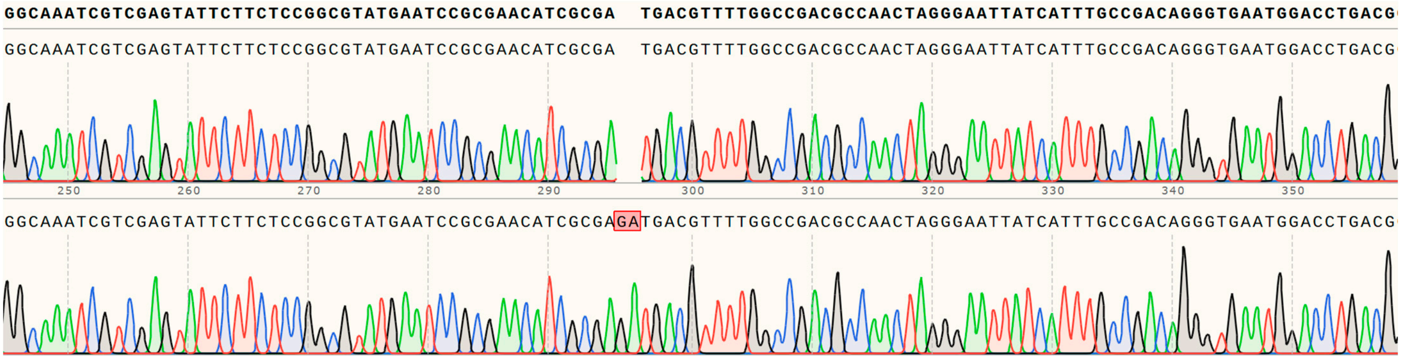

Figure S9. Comparing the sequences of *Os10g0174548* of H574 (WT) and H580. The insertion occurred at the 5,164,390 bp on chromosome 10.

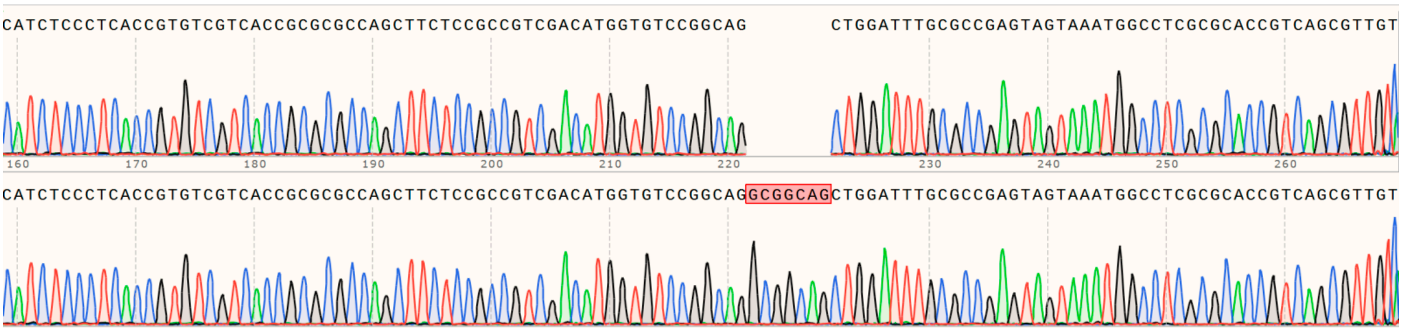

Figure S10. Comparing the sequences of *Os10g0163290* of H574 (WT) and H580. The insertion occurred at the 4,326,731 bp on chromosome 10.

Table S1. Summary of mutation information in high frequency mutation regions

Line	Position	Mutation rate in HF	Mutation rate of the corresponding chromosome	Number of mutation		
				Total	SBSs	InDels
MP1	Chr.2(16~ 18Mb)	2.4×10^{-3}	4.3×10^{-5}	5,480	4,903	577
MP1	Chr.6(3~ 5Mb)	2.2×10^{-3}	7.7×10^{-5}	4,348	3,700	648
MP1	Chr.12(9~ 17Mb)	3.3×10^{-3}	6.3×10^{-5}	26,291	23,927	2,364
MP2	Chr.3(16~22Mb)	2.2×10^{-3}	6.3×10^{-5}	13,279	12,128	1,151
MP3	Chr.7(4~9Mb)	3.3×10^{-3}	4.1×10^{-5}	16,378	14,196	2,182
MP3	Chr.3(8~10Mb)	2.4×10^{-3}	6.6×10^{-5}	4,805	4,131	674
MP3	Chr.10(4~6Mb)	1.8×10^{-3}	9.7×10^{-5}	3,651	3,324	327
